# Cytomegalovirus pulls strings behind NK cells

**DOI:** 10.18632/oncotarget.21377

**Published:** 2017-09-28

**Authors:** Norimitsu Kadowaki, Kenichi Ishiyama, Toshio Kitawaki

**Affiliations:** Norimitsu Kadowaki: Department of Internal Medicine, Division of Hematology, Rheumatology and Respiratory Medicine, Faculty of Medicine, Kagawa University, Kagawa, Japan

**Keywords:** dasatinib, Ph^+^ leukemia, cytomegalovirus, NK cells, principal component analysis

Tyrosine kinase inhibitors (TKIs) targeting the BCR-ABL1 kinase have revolutionized the treatment strategy for Philadelphia chromosome-positive (Ph^+^) chronic myelogenous leukemia (CML) and acute lymphoblastic leukemia (ALL). Among the TKIs, dasatinib is distinctive in that it inhibits a broad spectrum of tyrosine kinases, including key regulators of immune responses [1]. In agreement with this profile, dasatinib suppresses activity of various immune cells, such as T cells, NK cells, and plasmacytoid dendritic cells *in vitro*. Meanwhile, a unique immunological phenomenon, expansion of large granular lymphocytes (LGLs), has been frequently observed in Ph^+^ leukemia patients treated with dasatinib. Such expansion does not occur with other TKIs, imatinib or nilotinib. Importantly, LGL expansion during dasatinib treatment is associated with superior therapeutic responses [2]. Thus, elucidating the mechanisms underlying the LGL expansion is instrumental in improving the treatment outcome for Ph^+^ leukemia.

Here is an enigma; dasatinib is immunosuppressive *in vitro*, whereas it is immunostimulatory *in vivo*. How can we reconcile these apparently opposite phenomena? A previous study implicated cytomegalovirus (CMV) in the LGL expansion, since it is predominantly observed in CMV-seropositive (CMV^+^) patients [3]. However, CMV reactivation is observed only in a small fraction of patients, and therefore, underlying factors in the lymphocytosis were uncertain. Thus, we aimed to identify them.

LGLs are composed of CD8^+^ T cells, γδT cells, and NK cells, collectively cytotoxic lymphocytes. We first identified NK cells as the dominant LGLs expanding in dasatinib-treated patients. All the patients with NK cell expansion were CMV^+^. NK cells express a complex mosaic of inhibitory and activating receptors with large variation among individuals. In order to assess the accurate status of such variegated cells, we performed multiparametric phenotyping of NK cells from healthy donors and Ph^+^ leukemia patients treated with imatinib, nilotinib or dasatinib. We then analyzed the data using principal component analysis (PCA), a mathematical algorithm that reduces dimensionality of multiparametric data by defining a novel parameter (principal component) as a combination of the parameters to preserve the most essential characteristics of the dataset. PCA revealed that NK cells from CMV^+^ dasatinib-treated patients underwent phenotypic progression that reflects CMV-associated differentiation (such as NKG2C^high^ CD57^high^ LIR-1^high^ NKp30^low^ NKp46^low^). NK cells from CMV^+^ imatinib- or nilotinib-treated patients and CMV^+^ healthy donors had an intermediate phenotype, and those from CMV-seronegative individuals did not have the CMV-associated phenotype. Seven of 30 CMV^+^ dasatinib-treated patients developed CMV reactivation as defined by an elevation of CMV-IgM or a positive result of PCR. Notably, the CMV-associated highly differentiated status of NK cells was already observed at leukemia diagnosis in the majority of CMV^+^ patients, and was further enhanced after starting dasatinib in virtually all CMV^+^ patients. CMV reactivation was detected in 8 of 20 CMV^+^ patients at leukemia diagnosis. Importantly, the patients with a higher degree of NK cell differentiation at diagnosis underwent significantly greater NK cell expansion and faster decreases in *BCR-ABL1* mRNA after starting dasatinib. Thus, pretreatment differentiated status of NK cells predicts robust expansion of NK cells and an earlier clinical response after starting dasatinib in CMV^+^ patients.

**Figure 1:**
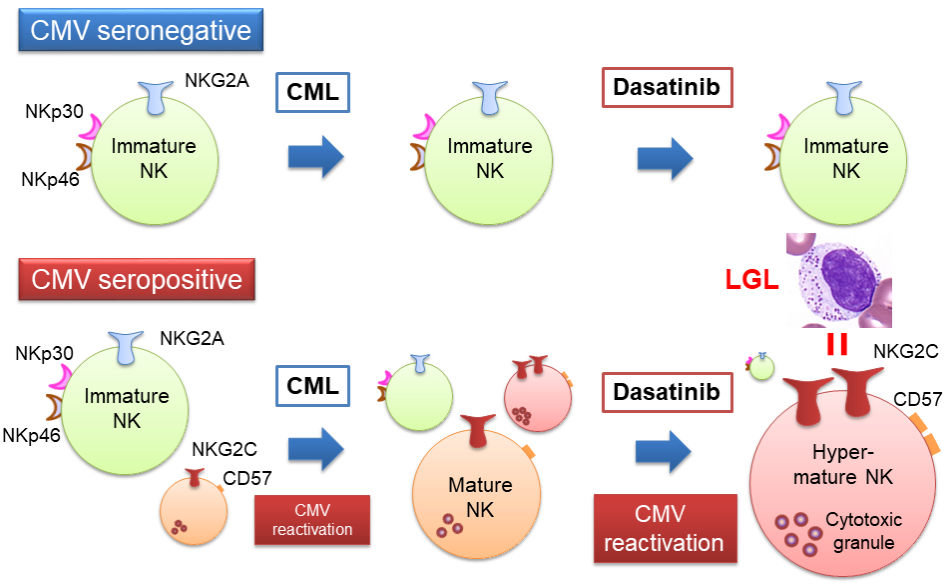
Mechanisms of NK cell expansion in CMV-seropositive patients treated with dasatinib In CMV-seronegative patients, CMV-associated NK cells are not observed during the whole course of CML onset and dasatinib treatment, and virtually all the NK cells are NKG2A^+^NKG2C^-^NKp30^high^NKp46^high^. In contrast, in CMV-seropositive patients, a small number of CMV-associated NKG2C^+^CD57^+^ NK cells have been generated. The CML onset appears to trigger subclinical CMV reactivation and consequent activation of the CMV-associated NK cells. Dasatinib treatment further reactivates CMV, leading to further activation and proliferation of the NK cells, which are recognized as LGLs.

In this study, the PCA revealed that virtually all the CMV^+^ patients exhibited enhancement of CMV-associated NK cell differentiation during dasatinib treatment, regardless of the presence or absence of documented CMV reactivation. We assume that a low level of continuous CMV reactivation, often below detection levels of assay, is responsible for the NK cell differentiation. Furthermore, similar enhancement of NK cell differentiation was observed with mere presence of leukemia, exclusively again in CMV^+^ patients. Based on these comprehensive analyses, we propose CMV as an initiation factor followed by leukemia and dasatinib as enhancing factors in the NK cell activation (Figure 1).

Notably, the CMV-associated NKG2C^+^ NK cells are considered to represent the human counterpart of mouse memory NK cells, and acquire distinctive molecular signature by epigenetic modification (FcRγ^-^ PLZF^-^ SYK^-^ EAT-2^-^) [4]. It is possible that the prompt expansion of NK cells with higher degrees of differentiation after starting dasatinib may reflect the property of immunological memory. Since such adaptive NK cells also appear to reduce relapse of hematological malignancies in allogeneic transplant patients who experienced CMV reactivation [5], the CMV-associated distinctive type of adaptive NK cells may contribute to suppressing leukemia relapse in transplant patients as well as in dasatinib-treated patients.

An important issue in CML treatment is whether TKIs can be discontinued after achieving a deep molecular response. A substantial proportion of such patients maintains the response for more than 12 months after discontinuation of TKIs. Notably, increases in NK cells [6, 7] but not T cells [7] associate with longer treatment-free remission, implying that immunological surveillance by NK cells is involved in keeping CML under control. Thus, the CMV-associated NK cell activation may contribute to preventing CML relapse after discontinuing dasatinib.

Our study using PCA suggests that the distinctive NK cell subset, that is, CMV-associated adaptive NK cells, is responsible for the dasatinib-induced NK cell expansion, which likely occurs through subclinical CMV reactivation. Intriguingly, the CMV-associated highly differentiated status is already observed at leukemia diagnosis in a large proportion of patients, and predicts prompt expansion of NK cells after starting dasatinib. These findings provide a rationale for the exploitation of CMV-associated NK cell activation to improve the treatment outcome for Ph^+^ leukemia. Furthermore, the findings may illustrate exploitation of immunity against resident viruses, virobiota, for the control of malignancies [8].
